# Accurate detection of Newcastle disease virus using proximity‐dependent DNA aptamer ligation assays

**DOI:** 10.1002/2211-5463.13117

**Published:** 2021-03-11

**Authors:** Boutheina Marnissi, Khouloud Khalfaoui, Tonge Ebai, Felipe Marques Souza de Oliveira, Abdeljelil Ghram, Masood Kamali‐Moghaddam, Issam Hmila

**Affiliations:** ^1^ Laboratory of Epidemiology and Veterinary Microbiology Institut Pasteur of Tunis University Tunis El Manar Tunis Tunisia; ^2^ Department of Immunology, Genetics and Pathology Science for Life Laboratory Uppsala University Sweden; ^3^ Present address: Magee‐Womens Research Institute Department of Obstetrics, Gynecology and Reproductive Sciences University of Pittsburgh Medical School PA USA

**Keywords:** aptamers, Newcastle disease virus, proximity ligation assays, rRT‐PCR, sandwich ELAA

## Abstract

Detecting viral antigens at low concentrations in field samples can be crucial for early veterinary diagnostics. Proximity ligation assays (PLAs) in both solution and solid‐phase formats are widely used for high‐performance protein detection in medical research. However, the affinity reagents used, which are mainly poly‐ and monoclonal antibodies, play an important role in the performance of PLAs. Here, we have established the first homogeneous and solid‐phase proximity‐dependent DNA aptamer ligation assays for rapid and accurate detection of Newcastle disease virus (NDV). NDV is detected by a pair of extended DNA aptamers that, upon binding in proximity to proteins on the envelope of the virus, are joined by enzymatic ligation to form a unique amplicon that can be sensitively detected using real‐time PCR. The sensitivity, specificity, and reproducibility of the assays were validated using 40 farm samples. The results demonstrated that the developed homogeneous and solid‐phase PLAs, which use NDV‐selective DNA aptamers, are more sensitive than the sandwich enzymatic‐linked aptamer assay (ELAA), and have a comparable sensitivity to real‐time reverse transcription PCR (rRT‐PCR) as the gold standard detection method. In addition, the solid‐phase PLA was shown to have a greater dynamic range with improved lower limit of detection, upper‐ and lower limit of quantification, and minimal detectable dose as compared with those of ELAA and rRT‐PCR. The specificity of PLA is shown to be concordant with rRT‐PCR.

## Graphical abstract

Newcastle disease virus is detected by a pair of extended DNA aptamers that, upon binding in proximity to proteins on the envelope of the virus, are joined by enzymatic ligation to form a unique amplicon that can be sensitively detected using real‐time PCR.

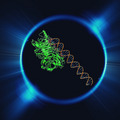

**Abbreviations**
Apt: aptamer
ELAA: Enzyme Linked Aptamer Assay
LLOQ: lower limit of quantification
LOD: limit of detection
MDD: minimal detectable dose
NDV: Newcastle Disease Virus
PLA: proximity ligation assay
rRT‐PCR: reverse real‐time PCR
SD: Standard deviation
SELEX: Systematic Evolution of Ligand by Exponential enrichment
ULOD: Upper limit of quantification

## Introduction

The World Organization for Animal Health has defined Newcastle disease (ND) as an infection of poultry with virulent strains of ND virus (NDV). This virus presents a perpetual threat to poultry, causing a high death rate during a very short time. The genome of NDV is linear, nonsegmented, single‐stranded RNA, encoding six proteins [1]. The hemagglutinin/neuraminidase (HN) and fusion (F) proteins are inserted in the envelope and represent the most important factor that determines the virulence and the infection cycle of the virus [2, 3]. HN is a multifunctional molecule that promotes the attachment of the virus to its sialic acid‐containing receptors, and hydrolyzes the sialic acid molecules from progeny viral particles to prevent viral self‐aggregation through the neuraminidase (NA) activity [4, 5]. In addition, HN is responsible for the membrane fusion through its interaction with the F protein, thereby facilitating the entry of viral RNA into the host cell [6, 7].

The hemagglutination (HA) followed by the hemagglutination inhibition (HI) tests is usually used to detect and identify NDV. These assays use red blood cells (RBCs) as indicators for the binding of the antigen to its antibody [8]. Besides, virus isolation in embryonated chicken eggs is a laborious and time‐consuming diagnostic method, which may take up to 2 weeks. Additionally, ELISA technique, which allows detection of highly abundant and specific viral proteins, is used for disease surveillance and/or monitoring.

Currently, NDV detection is conducted primarily at specialized diagnostic or reference laboratories, employing nucleic acid‐based methods including cDNA amplification and sequencing [9]. Compared to virus isolation, the nucleic acid‐based methods allow more rapid diagnosis. However, specific handling and transports of potentially infectious materials are required, and the integrity of the unstable RNA genome of the virus must be preserved to avoid false negative results. Application of the proximity ligation assays (PLAs) as a proteomic method for microbial detection [10] holds a great promise because of its specificity and superior detection sensitivity, as compared to PCR‐based diagnostic techniques, besides avoiding the need for genome extraction of targeted infectious agents. Hence, PLA‐based methods may provide suitable means for powerful veterinary diagnostic settings.

In previous publications, PLA was established using a pair of DNA aptamers as affinity probes to detect the target proteins [11]. The DNA aptamers were extended with additional DNA oligonucleotide sequences to form PLA probes. Upon binding of the DNA aptamers in proximity of the target protein, the two extended DNA sequences would hybridize to a common connector DNA oligonucleotide, allowing the ends to be joined covalently by enzymatic ligation. The ligation products were then amplified and quantified using real‐time PCR, while unreacted probes remain unamplified and undetectable. In this manner, conventional detection of protein molecules or infectious agents with limited sensitivity by other methods such as ELISA may be replaced by a highly sensitive and specific detection of reporter DNA molecules, using real‐time PCR [12]. To date, few examples of PLA‐based tests for the detection of viral pathogens have been reported. For instance, in an early study, porcine parvovirus (PPV) was detected with high sensitivity and specificity [13], and later, PLA was used to detect foot‐and‐mouth disease virus [14].

There is a great need for further improvement of NDV detection by establishing a sensitive method for rapid identification of the virus and immediate preventive measures to curtail spread of the virus. We therefore have developed in this study, an assay for antigenic detection using aptamer‐based PLA. The assay was based on aptamers targeting the surface proteins of the intact virus. The assay was then validated in farm samples to detect NDV.

## Results

### Aptamer‐assisted proximity ligation assay concept

**Fig. 1:**
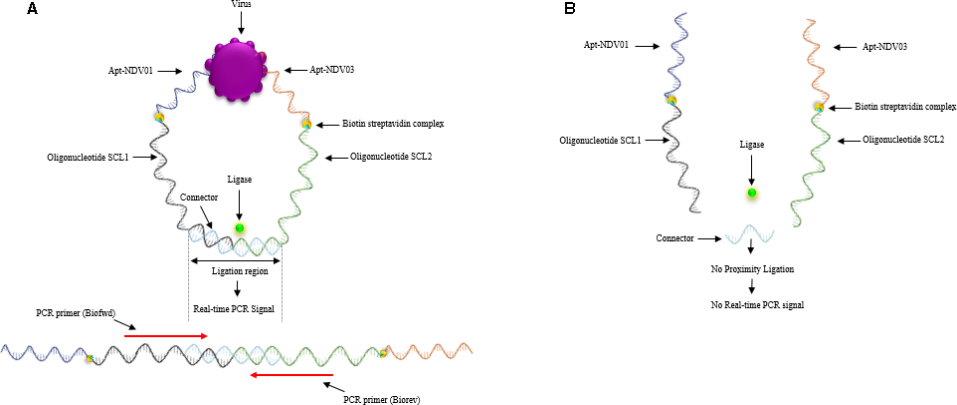
Illustration of PLA based on aptamers for NDV detection. (A) Two PLA probes that are bound to the same viral particle are in close proximity, which is resulted in a ligation‐dependent qPCR amplification. (B) Unbound probes in a sample lacking NDV are not in proximity, no ligation, and hence, no amplifiable DNA template is formed.

The conception of the PLA based on aptamers is illustrated in Fig. 1. The PLA probes were constructed by connecting the two biotinylated aptamers against the NDV [15] to two streptavidin‐conjugated DNA oligonucleotides via biotin‐streptavidin interaction. The DNA oligonucleotides have previously been designed and empirically evaluated for use in PLA [16]. In the presence of the NDV in the sample, the two aptamer‐based PLA probes bind to their target epitopes on the surface of the viral particle, allowing hybridization of a DNA connector oligonucleotide and a subsequent enzymatic DNA ligation of the free ends of the extended aptamers (Fig. 1). The newly formed DNA molecule is then used as template for signal amplification using real‐time quantitative PCR. In the absent of the viral particles, however, the PLA probes will not be in close proximity and no amplifiable DNA template is formed [11]. The numbers of PCR product are directly proportional to the ligated DNA amplicons reflecting the concentration of the NDV in the sample.

### Performance of aptamer‐assisted proximity ligation assay

The binding affinity, specificity, and compatibility of selected aptamers were described in our previous work [15]. To determine the limit of detection (LOD) of the sandwich Enzyme Linked Aptamer Assay (ELAA) test, LaSota vaccine strain titrating 10^6^ EID_50_·mL^−1^ was used, demonstrating that using such aptamers, the sandwich ELAA was able to detect as low as 1.2 (EID_50_·mL^−1^), as compared to the gold standard for NDV detection, the reverse real‐time PCR (rRT‐PCR) that provides a LOD of 0.6 (EID_50_·mL^−1^), when using the aptamers as affinity reagents.

**Fig. 2:**
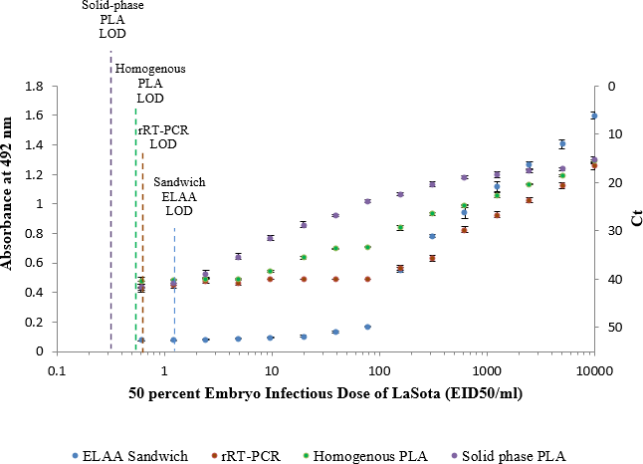
Determination of the sensitivity and the performance of the developed homogenous and solid‐phase PLA. The LLOQ, the ULOQ, the MDD, and the dynamic range were calculated for each test in addition to the LOD, which is defined as the concentration of viral protein detected at three SDs over the background.

We used the selected aptamers to establish PLA‐based tests for detection of NDV in farm samples. Both homogenous‐ and solid‐phase PLAs were utilized for highly sensitive and specific detection of NDV. The LOD of homogeneous PLA was 0.58 (EID_50_·mL^−1^), while that for solid‐phase PLA was slightly lower with 0.4 (EID_50_·mL^−1^). The reproducibility and the sensitivity of the PLA‐based tests were compared to those of sandwich ELAA and rRT‐PCR using data of triplicate reactions. The results revealed that solid‐phase PLA with a LOD of 0.4 (EID_50_·mL^−1^) is three times more sensitive than the sandwich ELAA and one and a half times more than rRT‐PCR and homogenous PLA (Fig. 2 and Table 1).

**Table 1:** Comparison of the LOD, the LLOQ, the ULOD, the MDD, and the dynamic range between sandwich ELAA, RT‐PCR, and solid‐phase and homogenous PLAs for the detection of NDV. The values presented in the table are the intersection of the calculated values as described in Fig. 2 with the abscissa axis.

	Sandwich ELAA	rRT‐PCR	Homogeneous PLA	Solid‐phase PLA
LOD (EID_50_·mL^−1^)	1.2	0.6	0.58	0.4
LLOQ (EID_50_·mL^−1^)	20	0.5	0.3	0.1
ULOD (EID_50_·mL^−1^)	7^3^	10^3^	10^4^	10^5^
MDD (EID_50_·mL^−1^)	1	0.2	0.2	0.1
Dynamic range	10^3^	10^5^	10^7^	10^8^

The results showed that homogeneous and solid‐phase PLAs were more sensitive than sandwich ELAA and rRT‐PCR. Furthermore, the solid‐phase PLA demonstrated greater dynamic range with better values of LOD, the lower limit of quantification (LLOQ), the upper limit of quantification (ULOD), and the minimal detectable dose (MDD), as compared to the other tests.

### Detection of NDV in farm samples

To test the applicability of the PLA tests with the selected aptamers as affinity binders for farm samples, 40 nasal per cloacal swabs were collected and analyzed using sandwich ELAA, homogenous‐ and solid‐phase PLAs. The sensitivity and specificity of developed methods were compared to those obtained by rRT‐PCR test as the gold standard. The results are summarized in Table 2. The sensitivity was calculated as sensitivity = TP/(TP + FN), whereas the specificity was calculates as specificity = TN/(TN + FP), where TP = true positive; FP = false positive; TN = true negative; FN = false negative.

**Table 2:** Sensitivity and specificity of homogeneous‐ and solid‐phase PLA, and Sandwich ELAA compared to rRT‐PCR test for the detection of NDV in 40 farm samples. The sensitivity = TP/(TP + FN); the specificity = TN/(TN + FP); TP = true positive (+/+); FP = false positive (−/+); TN = true negative (−/−); FN = false negative (+/−).

Test	Number of positives and negatives samples				Concordance	Sensitivity		Specificity	
Sandwich ELAA	+/+	+/−	−/+	−/−	%	TP/(TP + FN)	%	TN/(TN + FP)	%
Homogeneous PLA	26	0	0	14	100	26/26	100	14/14	100
Solid‐phase PLA	26	0	0	14	100	26/26	100	14/14	100
rRT‐PCR	26	0	0	14	100	26/26	100	14/14	100

The sandwich ELAA and the PLA tests, developed in this study, successfully detected 26 NDV positive samples out of 40 tested swabs. The results were statistically significant (*P* < 0.01). The obtained results for sandwich ELAA and for the PLA tests were 100% concordant with rRT‐PCR. Detailed analysis of the results relative to the diagnosis of NDV in farm samples are presented in the Tables S1 and S2.

**Fig. 3:**
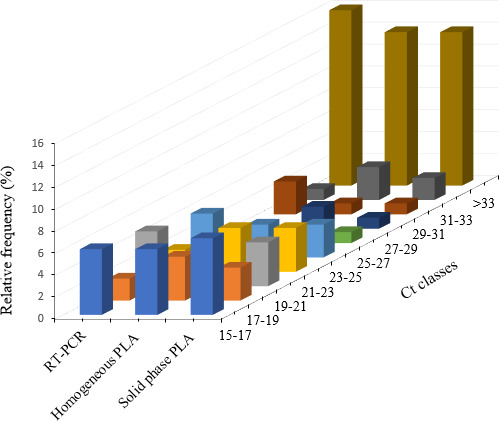
Distribution frequencies of Ct values for a set of 40 farm samples analyzed by rRT‐PCR, homogenous‐ and solid‐phase PLAs for the detection of NDV. The sensitivity of the PLA tests in both homogenous and solid‐phase formats were investigated and compared to that of rRT‐PCR. The results showed that the newly developed solid‐phase PLA exhibited higher analytical sensitivity.

The distribution frequencies of obtained Ct values for the 40 farm samples tested by PLAs and rRT‐PCR are displayed in Fig. 3.

## Discussion

Sensitive techniques for detecting viral surface proteins or nucleic acids are critical for early and accurate NDV diagnosis. Antibody‐based techniques, such as ELISA and hemagglutination assay, have proven to be important tools for the detection of viral proteins. However, these methods require significant amounts of starting materials, and are vulnerable to nonspecific background signals, which may result in lower assay sensitivity. Besides, the production of antibodies is time‐consuming, and the accuracy of such antibody‐based assays depends on the quality of the used antibody batches. Nucleic acid‐based methods such as PCR assays are, highly sensitive, easier to establish, and usually more efficient than antibody‐based methods. However, DNA detection does not demonstrate viable infectious pathogens, RNA result does not provide information on protein functionality, and such tests usually require sample preparation for viral genome isolation and cDNA synthesis. Immuno‐PCR [17] and real‐time immuno‐qPCR [18] represent the first trials to incorporate strengths into both approaches, addressing the weaknesses associated with conventional immunoassays. Nonetheless, the sensitivity and the specificity of immuno‐PCR‐based assays remain limited and depend on antibody specificity. As for conventional ELISA, these methods are prone to high background signals [19] as cross‐reactive signals from single antibody binding to off‐target proteins may be amplified [20]. Thus, it is critical to develop a rapid, sensitive, accurate, and widely available diagnostic test for the detection of NDV. PLA is reported to be exquisitely sensitive technique that relies on real‐time PCR, with demonstrated lower background than other immuno‐PCR methods [21]. PLA utilizes a pair of DNA‐tagged aptamers or antibody to detect proteins, protein modifications or protein–protein interactions. This method may be conducted in both homogeneous‐ and solid‐phase formats, allowing accurate visualization of single protein molecules or complexes with higher precision. These properties underline the potential of PLA in research, diagnosis, pharmacology, and a wide variety of applications that require high specificity and sensitivity, and accurate protein expression assessments such as cancer biomarkers [22, 23], prions [24], exosomes [25, 26], and personalized medicine [24]. Furthermore, PLA has been reported to detect a range of viral disease agents such as foot‐and‐mouth disease virus [14]. However, to date, no study has been reported on the detection of NDV using aptamer technology.

Thus, we present the first homogeneous‐ and solid‐phase PLA, with DNA aptamers as affinity reagents, for the detection of NDV in farm samples. There are numerous advantages of using DNA aptamers in PLA as high‐affinity binders for sensitive target recognition, such as easy chemical production and modification process and reagent stability. In addition, synthetic DNA aptamers are inexpensive compared to antibody production, with no batch‐to‐batch variations. Because of their ability to bind tightly and specifically to their targets, aptamers are used in a variety of diagnostic and therapeutic assays [27].

Importantly, aptamers may prove useful for the detection of a wide variety of viruses ranging from detection of Influenza, Dengue, Ebola and SARS to NDV and SARS‐CoV2 [28]. The emergence of NDV and the increased rate of morbidity and mortality associated with it worldwide, make the establishment of accurate laboratory assays critical for poultry management and epidemic surveillance and NDV control. However, current diagnostic tests for NDV disease, including ELISA and rRT‐PCR, may be time‐consuming, and suffering from low sensitivity. By adopting selected DNA aptamers, based on Systematic Evolution of Ligand by Exponential enrichment (SELEX) technology and high‐throughput sequencing, we have explored the possibility of NDV detection using PLA that uses DNA aptamers in homogenous and solid‐phase formats. In this report, we demonstrate that PLA‐based test presents an analytical sensitivity similar, if not greater, to that of rRT‐PCR; thus, it can be suggested that this new methodology may be a rapid and reliable tool for the diagnosis of NDV, and presents an alternative to the ELISA‐based test. The homogeneous PLA allowed detection of as few as 0.58 (EID_50_·mL^−1^), which is similar to the analytical sensitivity of the rRT‐PCR‐based assay, and superior to that of the sandwich ELAA. Furthermore, the solid‐phase PLA with a LOD of 0.4 (EID_50_·mL^−1^) is one and a half times more efficient than the homogeneous PLA and the rRT‐PCR, and three times more sensitive than the Sandwich ELAA. The diagnostic sensitivity, specificity, and robustness of the PLAs are heavily dependent upon the suitability of the binding aptamers used in such assays. Both Apt_NDV01 and Apt_NDV03 were selected using LaSota vaccine as a target along with SELEX process combined with high‐throughput sequencing. The affinity, the specificity, and the compatibility of these two selected aptamers used in the sandwich assays have been evaluated in our previous study [15]. The binding analysis revealing that both aptamers recognize NDV in field clinical samples with high specificity and nanomolar affinities [15]. The developed PLA‐based tests for the detection of NVD were further evaluated on the farm samples previously analyzed by rRT‐PCR and sandwich ELAA. When these samples were analyzed by the PLA, a 100% concordance with the previously developed sandwich ELAA was demonstrated.

Compared to RT‐PCR, PLA exhibits several advantages. It is much simpler to perform and demonstrates higher diagnostic sensitivity with very low background noises. In addition, PLAs does not require any sample preparation step as only a dilution of the original sample is performed before being added to a mix of the proximity probes.

In summary, we report for the first time the use of aptamers in homogeneous‐ and solid‐phase PLAs to detect NDV and overcome limitations of laboriousness or lack of sensitivity of current assays for the detection of viral genomes and/or proteins. With such analytical sensitivity, similar or greater to that of rRT‐PCR, the PLAs are sensitive, rapid, reproducible, and reliable tools for the diagnosis of NDV in farm samples and could be an alternative to rRT‐PCR, with a clear potential for a multiplex application for the detection of various avian viruses.

## Materials and methods

### Reagents

Dynabeads M‐280 Streptavidin (10 mg·mL^−1^) were purchased from Thermo Fisher Scientific (Artenay, France). All biotinylated aptamers and other DNA oligonucleotides (Table 3) were purchased from RAN BIOLINKS (Tunis, Tunisia). Washing buffer was composed of 1× PBS (pH 7.2), 0.1% BSA, and 0.05% Tween‐20 (Sigma‐Aldrich, Taufkirchen, Germany). PLA buffer was composed of 1× PBS (pH 7.2), 0.1% BSA, 0.05% Tween‐20, 100 nm goat IgG, 0.1 µg·µL^−1^ salmon sperm DNA, and 5 mm EDTA. Probe storage buffer contained 1× PBS (pH 7.2), 0.1 % BSA and 0.05 % NaN_3_. Oligonucleotide storage buffer contained 1 mm Tris/HCl (pH 7.2) and 0.1 mm EDTA. All enzymes and dNTPs were purchased from New England Biolabs (Paris, France).

**Table 3:** List of DNA oligonucleotides.

Oligonucleotide		Sequence (5′–3′)	Modification	Reference
Aptamers	Apt_NDV01	5′‐GGGGTCTTGCAGGTCCCGTA GGAGGGGCCATTGGAGTGGGG‐3′	5′‐Biotin or 5′ DIG	[15]
	Apt_NDV03	5′‐CGATGGAGGACCTCCGGTTT ACCGTGTCGTTTTACTCTTG‐3′	5′‐Biotin or 5′ DIG	
Oligonucleotides	SCL1	5′‐CGCATCGCCCTTGGACTACGA CTGACGAACCGCTTTGCCTGACT GATCGCTAAATCGTG‐3′	5′‐SAV^a^	[32]
	SCL2	5′‐TCGTGTCTAAAGTCCGTTACC TTGATTCCCCTAACCCTCTTGAA AAATTCGGCATCGGT‐3′	5′‐Phosphate, SAV‐3′	
Ligation template	Connector oligo	5 ′ ‐TACTTAGACACGACACGATTT‐3 ′	None	
qPCR probe	TaqMan probe	5 ′ ‐TGACGAACCGCTTTGCTGA‐3 ′	5′‐FAM, MGB^b^‐3′	
qPCR primers	Biofwd (forward primer)	5 ′ ‐CATCGCCCTTGGACTACGA‐3 ′		
	Biorev (reverse primer)	5 ′ ‐GGGAATCAAGGTAACGGACTTTAG‐3 ′		
rRT‐PCR primers	forward	5 ′ ‐AGTGATTGTCTCGGACCTTC‐3 ′	None	[33]
	reverse	5 ′ ‐CCTGAGGAGAGGCATTTGCTA‐3 ′	None	
rRT‐PCR probe	TaqMan probe	5 ′ ‐TTCTCTAGCAGTGGGACAGCCTGC‐3 ′	5′‐Texas Red, BHQ‐2^c^‐3′	

^a^ Streptavidin.

^b^ Minor Groove Binder.

^c^ Black Hole Quencher.

### Farm samples

The study was performed on 40 poultry samples including tracheal (ET) and cloacal swabs (EC), as well as internal organs consisting of allantois (A), kidneys (K), lung (L), liver (Li), and trachea (T), collected from suspected chickens showing NDV clinical signs. They were collected from eight farms, located in the provinces of Bizerte, Nabeul, Ben Arous, Sidi Bouzid, Beja, Ariana, Sfax, and Jendouba, and sent rapidly to the diagnostic laboratory at the Institute Pasteur of Tunis for analysis. Field samples are sent by the public or private veterinarians in the frame of their routine follow‐up/monitoring of poultry farms, or when reporting a suspicious disease, especially for both NDV and avian influenza virus (AIV) infections, which are under continuous surveillance in Tunisia.

### Aptamers

Aptamer production and validation was previously described by Marnissi *et al*. [15]. Briefly, selection was performed using three rounds of systematic evolution of ligands by exponential enrichment (SELEX). Then, the highly enriched ssDNA pool was sequenced using quantitative high‐throughput sequencing, and the results were analyzed using FASTAptamer Toolkit. The reads were sorted by copy numbers and then clustered into families, according to their sequence homology. High‐frequency aptamer sequences were tested for their affinity and specificity, and further validated in a sandwich enzymatic‐linked aptamer assay (ELAA) for rapid detection of NDV in farm samples.

### Viral nucleic acid extraction

Viral RNA was extracted from LaSota vaccine strain and NDV present in collected farm samples, using TRIzol reagent (Invitrogen, Carlsbad, CA, USA), according to the manufacturer's instructions. The final extracted pellets were suspended in 20 μL RNase‐free water and stored at −80 °C.

### Sandwich ELAA

Sandwich ELAA was performed in Streptavidin‐coated 96‐well microtiter plates (Thermo Fisher Scientific), as follows: 100 µL of 10 nm Biotin‐Apt_NDV03 was denatured by heating at 95 °C for 5 min and cooled on ice for 10 min. The aptamers were added to a microtiter plate and incubated for 1 h at room temperature (RT). Then, the wells were washed three times with 200 µL of washing buffer. Typically, 1 μL of each sample aliquot was diluted in 100 μL 1× PBS and added into each well, and incubated for 1 h, at RT. Wells were washed three times, and 100 µL of 10 nm digoxigenin‐labeled Apt_NDV01 was added. After 1 h of incubation at RT and three washes, an anti‐digoxigenin antibody (1 : 2000) was added to each well and left to react for 30 min. The wells were washed three times, and a solution of OPD was added. Finally, the reaction was stopped with 2 NH_2_SO_4_ and the absorbance recorded at 492 nm.

### One‐step real‐time reverse transcription PCR

One‐step rRT‐PCR was conducted in a total volume of 15 μL, using AgPath‐ID™ One‐Step RT‐PCR Kit (Applied Biosystems TM, Artenay, France) by mixing 7.5 μL of 2× RT‐PCR Buffer, 0.6 µL of 25× RT‐PCR Enzyme Mix, 2 μL of RNA template, 0.3 μm of each forward and reverse primer specific to polymerase (M) gene, and 0.2 μm TaqMan™ (ThermoFisher, Artenay, France) probe and RNase‐free water to reach the final volume of 15 μL. The mixture was incubated at 45 °C for 10 min followed by 95 °C for 10 min and 45 cycles of 95 °C for 15 s and 60 °C for 45 s. A negative control, in which the samples were replaced by water, was included in each PCR run.

### Proximity ligation assays

#### PLA probe preparation and immobilization of aptamers on microparticles

The PLA probes were prepared using a slightly modified PLA protocol reported by Oliveira *et al*. [29]. Two PLA probes were prepared by mixing 10 µL of 100 nm SCL1 DNA oligonucleotide with 10 µL of 100 nm biotinylated Apt_NDV01, and 10 µL of 100 nm SCL2 DNA oligonucleotide with 10 µL of 100 nm biotinylated Apt_NDV03. Following 10 s spin at 21 062 ***g***, the mixtures were incubated at RT for 60 min. Then, 90 μL of probe storage buffer was added to the mixtures and the incubations were continued for 30 min at 20 °C. The probes may be kept at 4 °C for up to 6 months. Prior to their use, the aptamers were denatured by heating at 95 °C for 5 min and then cooled on ice for 10 min.

For the solid‐phase PLA, magnetic beads are used as solid support on which an aptamer is immobilized as capture affinity binder. For that, 100 μL of streptavidin‐coated Dynabeads (10 mg·mL^−1^) microparticles was washed three times with 500 μL of washing buffer. Then, the biotinylated Apt‐NDV01 was diluted to 50 nm in storage buffer from which 200 μL were denatured by heating at 95 °C for 5 min and cooled on ice for 10 min. The aptamer solution was then mixed with the magnetic beads, followed by incubation for 1 h at RT, with gentle shaking. The magnetic beads were collected on a magnet for 30 s, and the supernatant was discarded. The beads were then washed twice with 500 μL PLA buffer and resuspended in 200 μL of storage buffer. The conjugates may be kept at 4 °C for up to 6 months.

### Sample preparation

#### Homogenous PLA

An aliquot of 10^6^ EID_50_·mL^−1^ of LaSota vaccine strain, diluted in PLA buffer, was used to prepare a twofold serial dilution. For each PLA reaction, 45 μL of diluted sample was used.

The PLA probe mixture was prepared by diluting each PLA probe separately in the PLA buffer to 1 nm. Then, an equal volume of each probe was mixed in PLA buffer to a final concentration of 500 pm for each probe and incubated at RT for 5 min.

For each homogenous PLA reaction, 2 µL of PLA probe mix was added to 2 µL of each sample diluted with DMSO dilution buffer, and incubated for 2 h at RT. For clinical samples, a positive control made of 2 µL of diluted LaSota vaccine as well as a negative control, for which the sample was replaced by 2 µL of PBS/0.1% BSA, were included. After the incubation time, 2 μL of the mixture was added to 25 μL ligation/PCR mix (Table 4) and incubated for 5 min at RT. Then, qPCR was performed using an initial step of 95 °C for 2 min, followed by 40 cycles of 95 °C for 5 s and 60 °C for 30 s. All reactions were carried out in triplicate.

**Table 4:** Ligation and PCR mix.

Reagents	Stock concentration	Volume (mL)	Final concentration
PCR buffer	10×	2.5	1×
MgCl_2_	50 mm	1.25	2.5 mm
TaqMan probe	10 μm	0.6	0.22 μm
Biofwd	10 μm	0.25	0.1 μm
Biorev	10 μm	0.25	0.1 μm
Biosplint	10 μm	0.25	0.1 μm
ATP	100 mm	0.02	0.08 mm
dN (A,C,G,U)TP	25 mm	0.2	0.2 mm
Taq polymerase	5 U·μL^−1^	0.15	0.03 U·μL^−1^
T4DNA ligase	1 U·μL^−1^	0.25	0.01 U·μL^−1^
UDG	1 U·μL^−1^	0.05	0.002 U·μL^−1^
H_2_O		19.23	
Total volume (mL)		25	

#### Solid‐phase PLA

Previously prepared Apt‐NDV 01‐coated magnetic beads were vortexed to a homogeneous suspension, and 1 μL was pipetted into a 1.5‐mL tube. The storage buffer was discarded, and the beads were mixed with 5 μL of PLA buffer. Subsequently, the bead suspension was mixed with 45 μL of diluted samples in PCR strip; the mixture was shortly vortexed and incubated for 1 h at RT under rotation. The microparticles were washed three times, 50 μL PLA probe mix (250 pm of each probe) was added, and the mixture was incubated for 1 h at RT under rotation. The beads were washed twice, and then, 25 µL of ligation and PCR mix (Table 4) were added to the beads, and the qPCR was performed as described above. For each run, a positive control containing diluted LaSota vaccine and a negative control made of PBS, 0.1% BSA were included. All washing steps were done using the DynaMag™‐Spin Magnet (ThermoFisher Scientific, USA).

### Statistics

The one‐way ANOVA test, using Simple Inter‐active Statistical Analysis online tool (http://www.quantitativeskills.com/sisa/index.htm), was used to perform statistical analyses. The results were defined as significantly different if *P* < 0.01. The one‐way ANOVA test was also used to calculate the 95% confidence intervals (CI) of each mean.

The mean and the standard deviation (SD), the coefficient of variation percentage (CV%) of the optical densities (OD 492 nm) for the sandwich ELAA, the threshold cycle (Ct) of rRT‐PCR, and PLAs were further analyzed using Excel software. For rRT‐PCR and qPCR readouts of cutoff points were fixed empirically at 40 Ct [30].

The relative variability (CV %) between triplicates of one sample was calculated as CV % = (SD_sample triplicates_/Mean_sample triplicates_)*100 and was defined as significantly different, if CV % < 20%.

statpluspro version 5.9.8 was used to calculate the LOD [31], the LLOQ, the ULOQ, the MDD, and the dynamic range of each method, as described by Marnissi *et al*. [15]. Briefly, the LOD was calculated as LOD = background signal + (3 × SD_mean_), where the background signal corresponds to the mean value of three negative control samples. The LLOQ is the lowest concentration at which the product can be accurately identified and at which certain predefined bias and imprecision targets are achieved. The LLOQ was determined as LLOQ = LOD + (10 × SD_background_). The upper ULOQ was determined as ULOQ = *f* (*X* − (3 × SD_X_), and the MDD was determined as MDD = 2 × SD_background mean_. The assay sensitivity was calculated as sensitivity = TP/(TP + FN), whereas the assay specificity was calculated as specificity = TN/(TN + FP), where TP = true positive; FP = false positive; TN = true negative; FN = false negative.

## Conflict of interest

The authors declare no conflict of interest.

## Author contributions

BM and MK‐M designed the study; BM performed the experiments; BM analyzed the data; BM, KK, TE, FMSO, and MK‐M contributed with analysis tools/reagents; AG, MK‐M, and IH supervised and validated the data; BM wrote the manuscript; MK‐M, IH, and AG revised the manuscript. All authors reviewed and approved the final version of the manuscript.

## Supporting information


**Table S1.** Determination of 95% confidence intervals (CI) and coefficient of variability. The 95% confidence interval (CI) of each mean was calculated using the one‐way ANOVAs test. Therefore, a range between upper and lower numbers calculated from a sample was determined. The relative variability between triplicates of each sample was defined as significantly different if CV% < 20% using the same statistical test.

**Table S2.** Clinical performance of Homogeneous PLA, Solid‐phase PLA, Sandwich ELAA and rRT‐PCR tests. Results of the diagnosis of NDV in tracheal (ET) and cloacal swabs (EC) and internal organs, consisting of allantois (A), kidneys (K), lung (L), liver (Li) and trachea (T) collected from 40 chickens with suspected NDV infection. Results of Homogeneous PLA, Solid‐phase PLA, Sandwich ELAA and rRT‐PCR tests are reported as positive or negative for each sample.

## Data Availability

The data that support the findings of this study are available in the supplementary material of this article.
