# Supplementary material for: Accurate detection of Newcastle disease virus using proximity‐dependent DNA aptamer ligation assays
Source: FEBS Open Bio. 2021 Mar 11;11(4):1122–31. doi: 10.1002/2211-5463.13117 (PMC8016122; doi:10.1002/2211-5463.13117)
Supplement: Supplementary file 1 — Table S1. Determination of 95% confidence intervals (CI) and coefficient of variability. The 95% confidence interval (CI) of each mean was calculated using the one‐way ANOVAs test. Therefore, a range between upper and lower numbers calculated from a sample was determined. The relative variability between triplicates of each sample was defined as significantly different if CV% < 20% using the same statistical test. [file FEB4-11-1122-s001.docx]

**Supplementary Table S1. Determination of 95% confidence intervals (CI) and coefficient of variability**

The 95% confidence interval (CI) of each mean was calculated using the one-way ANOVAs test. Therefore, a range between upper and lower numbers calculated from a sample was determined. The relative variability between triplicates of each sample was defined as significantly different if CV% < 20 % using the same statistical test.

|  | Sandwich ELAA (OD) | | | rRT-PCR (Ct) | | | Homogeneous PLA (Ct) | | | Solid phase PLA (Ct) | | |
| --- | --- | --- | --- | --- | --- | --- | --- | --- | --- | --- | --- | --- |
| Samples | CV% | 95% t-C.I | | CV% | 95% t-C.I | | CV% | 95% t-C.I | | CV% | 95% t-C.I | |
| LaSota strain | 6.548 | 1.494 | 2.075 | 1.562 | 24.717 | 25.283 | 2.437 | 14.718 | 16.615 | 1.903 | 14.84 | 15.493 |
| 244/14(EC) | 7.917 | 1.126 | 1.677 | 0.5 | 19.887 | 20.113 | 3.151 | 17.789 | 20.811 | 1.881 | 18.269 | 19.064 |
| 289/14(EC) | 7.311 | 0.987 | 1.426 | 1.694 | 18.636 | 19.364 | 2.971 | 17.165 | 19.901 | 0.588 | 16.887 | 17.113 |
| 163/15(EC) | 12.876 | 0.066 | 0.128 | 0.292 | 40.864 | 41.136 | 0.372 | 40.654 | 41.413 | 1.833 | 40.802 | 42.531 |
| 540/15(ET) | 1.584 | 0.734 | 0.795 | 2.521 | 34.001 | 35.999 | 0.606 | 32.503 | 33.497 | 0.555 | 31.004 | 31.396 |
| 518/15(ET) | 5.508 | 0.621 | 0.818 | 0.524 | 32.804 | 33.196 | 1.265 | 31.333 | 33.367 | 0.837 | 31.301 | 31.899 |
| 546/15(ET) | 0.318 | 1.472 | 1.496 | 2.165 | 15.608 | 16.392 | 1.502 | 14.793 | 15.94 | 0.956 | 14.92 | 15.247 |
| 79/15(ET) | 3.471 | 1.326 | 1.577 | 1.082 | 15.804 | 16.196 | 1.392 | 15.109 | 16.191 | 0.569 | 15.102 | 15.298 |
| 538/15(A) | 17.907 | 0.065 | 0.169 | 0.481 | 41.274 | 41.726 | 0.362 | 41.787 | 42.546 | 1.024 | 41.76 | 42.74 |
| 286/15(ET) | 2.974 | 0.953 | 1.105 | 1.333 | 29.547 | 30.453 | 0.868 | 28.342 | 29.592 | 0.769 | 26.831 | 27.302 |
| 169/15(A) | 2.468 | 1.103 | 1.247 | 2.851 | 22.258 | 23.742 | 1.037 | 21.693 | 22.84 | 1.904 | 20.547 | 21.453 |
| 534/15(ET) | 3.124 | 1.19 | 1.391 | 3.5 | 19.208 | 20.792 | 0.79 | 18.954 | 19.713 | 1.477 | 19.207 | 19.86 |
| 556/15(EC) | 11.566 | 0.588 | 1.062 | 0.882 | 33.661 | 34.339 | 1.503 | 32.216 | 34.717 | 0.785 | 31.749 | 32.318 |
| 50/16(K+Li) | 11.764 | 0.06 | 0.109 | 0.615 | 42.701 | 43.299 | 0.462 | 42.703 | 43.697 | 0.681 | 42.007 | 42.66 |
| 65/16(K+T) | 15.433 | 0.06 | 0.136 | 0.478 | 41.574 | 42.026 | 0.701 | 40.45 | 41.884 | 0.786 | 40.503 | 41.23 |
| 100/16(K+T) | 3.025 | 0.889 | 1.034 | 2.603 | 29.116 | 30.884 | 0.544 | 27.654 | 28.413 | 0.463 | 24.803 | 25.064 |
| 003/16(K+T) | 13.067 | 0.049 | 0.096 | 0.692 | 41.34 | 41.993 | 0.69 | 41.116 | 42.55 | 0.684 | 41.84 | 42.493 |
| 29/16(K+T) | 17.364 | 0.048 | 0.123 | 1.211 | 40.964 | 42.103 | 0.777 | 40.568 | 42.165 | 1.744 | 40.648 | 42.285 |
| 64/16(EC) | 7.15 | 0.06 | 0.087 | 0.465 | 42.774 | 43.226 | 1.03 | 41.217 | 43.383 | 0.975 | 40.547 | 41.453 |
| 92/17(ET) | 3.948 | 0.06 | 0.073 | 1.091 | 41.481 | 42.519 | 0.87 | 40.504 | 42.296 | 0.418 | 41.204 | 41.596 |
| 134/17(K+T) | 1.703 | 1.124 | 1.223 | 1.357 | 30.196 | 31.138 | 0.845 | 29.142 | 30.392 | 1.306 | 28.538 | 29.395 |
| 148/17(L+T) | 0.3395 | 1.168 | 1.187 | 1.0175 | 25.701 | 26.299 | 1.147 | 24.45 | 25.884 | 0.718 | 23.904 | 24.296 |
| 174/17(L+T) | 0.606 | 1.136 | 1.171 | 1.2 | 24.661 | 25.339 | 0.809 | 24.203 | 25.19 | 2.16 | 22.275 | 23.392 |
| 31/17(L+T) | 9.89 | 0.068 | 0.113 | 1.279 | 42.87 | 44.13 | 0.496 | 41.416 | 42.45 | 0.87 | 40.992 | 41.808 |
| 81/17(L+T) | 15.342 | 0.058 | 0.129 | 1.213 | 40.108 | 41.225 | 1.029 | 39.399 | 41.468 | 0.922 | 40.985 | 41.849 |
| 83/17(L) | 15.626 | 0.054 | 0.124 | 0.733 | 41.321 | 42.012 | 1.374 | 39.954 | 42.779 | 0.485 | 42.598 | 43.069 |
| 147/17(L+T) | 10.846 | 0.062 | 0.108 | 0.983 | 41.862 | 42.804 | 0.588 | 42.108 | 43.358 | 0.673 | 42.507 | 43.16 |
| 29/18 (ET) | 3.444 | 1.586 | 1.883 | 1.785 | 15.45 | 16.884 | 3.296 | 14.292 | 16.841 | 1.009 | 14.96 | 15.306 |
| 106/18 (EC) | 2.1589 | 1.847 | 2.056 | 1.058 | 14.784 | 15.583 | 0.380 | 15.101 | 15.232 | 0.766 | 14.936 | 15.197 |
| 108/18 (ET) | 3.293 | 1.368 | 1.612 | 0.634 | 20.521 | 21.179 | 1.419 | 20.007 | 20.66 | 1.467 | 19.34 | 19.993 |
| 109/18 (ET) | 0.689 | 1.782 | 1.844 | 1.648 | 14.642 | 15.892 | 1.648 | 14.982 | 15.551 | 0.653 | 15.187 | 15.413 |
| 116/18 (ET) | 0.888 | 1.375 | 1.437 | 1.292 | 21.616 | 23.05 | 0.696 | 21.76 | 22.106 | 0.953 | 21.598 | 22.069 |
| 134/18 (ET) | 1.254 | 1.726 | 1.837 | 1.599 | 15.108 | 16.358 | 2.051 | 15.303 | 16.03 | 1.882 | 15.007 | 15.66 |
| 162/18 (EC) | 0.583 | .1862 | 1.221 | 1.527 | 22.704 | 24.496 | 1.273 | 22.34 | 22.993 | 2.074 | 21.744 | 22.789 |
| 163/18 (EC) | 1.89 | 1.416 | 1.556 | 2.793 | 9.232 | 22.101 | 1.553 | 19.321 | 20.012 | 1.041 | 18.974 | 19.426 |
| 215/18(ET) | 0.897 | 1.529 | 1.599 | 1.37 | 18.643 | 19.957 | 2.258 | 17.962 | 18.904 | 0.555 | 17.887 | 18.113 |
| 269/18 (EC) | 0.254 | 1.192 | 1.207 | 1.527 | 22.704 | 24.496 | 0.988 | 23.105 | 23.62 | 0.514 | 22.303 | 22.564 |
| 311/18 (ET) | 0.495 | 1.219 | 1.249 | 2.2 | 23.665 | 26.401 | 1.054 | 24.801 | 25.399 | 2.477 | 23.975 | 25.358 |
| 349/18 (ET) | 7.37 | 0.076 | 0.111 | 0.371 | 40.787 | 41.546 | 0.681 | 42.007 | 42.66 | 0.403 | 42.704 | 43.096 |
| 33/19 (EC) | 6.643 | 0.078 | 0.109 | 0.478 | 41.303 | 42.297 | 0.554 | 41.372 | 41.895 | 1.12 | 40.711 | 41.756 |
| 34/19(EC) | 1.295 | 1.195 | 1.274 | 1.428 | 20.661 | 21.339 | 0.952 | 20.774 | 21.226 | 1.275 | 19.449 | 20.018 |
| NC | 4.545 | 0.058 | 0.073 | 0.858 | 41.592 | 42.408 | 0.618 | 41.264 | 44.336 | 0.673 | 42.507 | 43.16 |
