# Supplementary material for: Accurate detection of Newcastle disease virus using proximity‐dependent DNA aptamer ligation assays
Source: FEBS Open Bio. 2021 Mar 11;11(4):1122–31. doi: 10.1002/2211-5463.13117 (PMC8016122; doi:10.1002/2211-5463.13117)
Supplement: Supplementary file 2 — Table S2. Clinical performance of Homogeneous PLA, Solid‐phase PLA, Sandwich ELAA and rRT‐PCR tests. Results of the diagnosis of NDV in tracheal (ET) and cloacal swabs (EC) and internal organs, consisting of allantois (A), kidneys (K), lung (L), liver (Li) and trachea (T) collected from 40 chickens with suspected NDV infection. Results of Homogeneous PLA, Solid‐phase PLA, Sandwich ELAA and rRT‐PCR tests are reported as positive or negative for each sample. [file FEB4-11-1122-s002.docx]

**Table S2. Clinical performance of Homogeneous PLA, Solid-phase PLA, Sandwich ELAA and rRT-PCR tests.**

Results of the diagnosis of NDV in tracheal (ET) and cloacal swabs (EC) and internal organs, consisting of allantois (A), kidneys (K), lung (L), liver (Li) and trachea (T) collected from 40 chickens with suspected NDV infection. Results of Homogeneous PLA, Solid-phase PLA, Sandwich ELAA and rRT-PCR tests are reported as positive or negative for each sample.

|  | Sandwich ELAA (OD) | | | | rRT-PCR (Ct) | | | |  | | Homogeneous PLA (Ct) | | | | | Solid-phase PLA (Ct) | | | | |
| --- | --- | --- | --- | --- | --- | --- | --- | --- | --- | --- | --- | --- | --- | --- | --- | --- | --- | --- | --- | --- |
| Samples | **Mean**  **OD** | **SD** | ***p*-value** | **Positive/**  **negative** | **Mean**  **Ct** | **SD** | ***p*-value** | **Positive/**  **negative** | | **Mean**  **Ct** | | **SD** | ***p*-value** | **Positive/**  **negative** | **Mean**  **Ct** | | **SD** | ***p*-value** | **Positive/**  **negative** |  |
| LaSota strain | 1.785 | 0.116 | 1.0E-5 | **Positive** | 16 | 0.25 | 0.0002 | **Positive** | | 15.666 | | 0.381 | 0.0002 | **Positive** | 15 | | 0.288 | 0.0001 | **Positive** |  |
| 244/14 (EC) | 1.402 | 0.111 | 3.0E-5 | **Positive** | 20 | 0.1 | 0.0001 | **Positive** | | 19.3 | | 0.608 | 0.0005 | **Positive** | 18.666 | | 0.351 | 0.0001 | **Positive** |  |
| 289/14 (EC) | 1.207 | 0.088 | 2.0E-5 | **Positive** | 19 | 0.321 | 0.0001 | **Positive** | | 18.533 | | 0.55 | 0.0004 | **Positive** | 17 | | 0.1 | 0 | **Positive** |  |
| 163/15 (EC) | 0.097 | 0.012 | 0.013 | Negative | 41 | 0.12 | 0.0103 | Negative | | 41.03 | | 0.152 | 0.022 | Negative | 41.66 | | 0.763 | 0.08 | Negative |  |
| 540/15 (ET) | 0.765 | 0.012 | 0.0001 | **Positive** | 35 | 0.882 | 0.004 | **Positive** | | 33 | | 0.2 | 0.0009 | **Positive** | 31.2 | | 0.173 | 0.0002 | **Positive** |  |
| 518/15 (ET) | 0.720 | 0.039 | 1.0E-5 | **Positive** | 33 | 0.173 | 0 | **Positive** | | 32.35 | | 0.409 | 0.0014 | **Positive** | 31.6 | | 0.264 | 0.0004 | **Positive** |  |
| 546/15 (ET) | 1.485 | 0.004 | 0 | **Positive** | 16 | 0.346 | 0.0001 | **Positive** | | 15.366 | | 0.23 | 0.0001 | **Positive** | 15.083 | | 0.144 | 0 | **Positive** |  |
| 79/15 (ET) | 1.452 | 0.05 | 0.0002 | **Positive** | 16 | 0.173 | 0 | **Positive** | | 15.65 | | 0.217 | 0.0001 | **Positive** | 15.2 | | 0.086 | 0 | **Positive** |  |
| 538/15 (A) | 0.117 | 0.02 | 0.0139 | Negative | 41.5 | 0.2 | 0.1036 | Negative | | 42.166 | | 0.152 | 0.1227 | Negative | 42.25 | | 0 | 0.1396 | Negative |  |
| 286/15 (ET) | 1.029 | 0.03 | 0.0002 | **Positive** | 30 | 0.4 | 0.0007 | **Positive** | | 28.96 | | 0.251 | 0.0005 | **Positive** | 27.06 | | 0.208 | 0.0002 | **Positive** |  |
| 169/15 (A) | 1.175 | 0.029 | 0.0001 | **Positive** | 23 | 0.655 | 0 | **Positive** | | 22.266 | | 0.23 | 0.0002 | **Positive** | 21 | | 0.4 | 0.0002 | **Positive** |  |
| 534/15 (ET) | 1.291 | 0.040 | 0.0002 | **Positive** | 20 | 0.7 | 0 | **Positive** | | 19.333 | | 0.152 | 0.0001 | **Positive** | 19.533 | | 0.288 | 0.0001 | **Positive** |  |
| 556/15 (EC) | 0.825 | 0.09 | 0.00016 | **Positive** | 34 | 0.3 | 1.0E-5 | **Positive** | | 33.466 | | 0.503 | 0.0023 | **Positive** | 32.033 | | 0.251 | 0.0004 | **Positive** |  |
| 50/16 (K+Li) | 0.085 | 0.01 | 0.034 | Negative | 43 | 0.26 | 0.0179 | Negative | | 43.2 | | 0.2 | 0.217 | Negative | 42.33 | | 0.28 | 0.05 | Negative |  |
| 65/16 (K+T) | 0.098 | 0.015 | 0.022 | Negative | 41.8 | 0.2 | 0.4481 | Negative | | 41.166 | | 0.288 | 0.0118 | Negative | 40.866 | | 0.3214 | 0.0152 | Negative |  |
| 100/16 (K+T) | 0.962 | 0.029 | 0.0002 | **Positive** | 30 | 0.781 | 2.0E-5 | **Positive** | | 28.033 | | 0.152 | 0.0003 | **Positive** | 24.933 | | 0.115 | 0.0001 | **Positive** |  |
| 003/16 (K+T) | 0.073 | 0.009 | 0.292 | Negative | 41.66 | 0.288 | 0.2794 | Negative | | 41.83 | | 0.288 | 0.0429 | Negative | 42.166 | | 0.288 | 0.0237 | Negative |  |
| 29/16 (K+T) | 0.086 | 0.014 | 0.085 | Negative | 41.533 | 0.503 | 0.261 | Negative | | 41.366 | | 0.321 | 0.0189 | Negative | 41.466 | | 0.723 | 0.0587 | Negative |  |
| 64/16 (EC) | 0.074 | 0.005 | 0.08498 | Negative | 43 | 0.2 | 0.0136 | Negative | | 42.3 | | 0.435 | 0.2415 | Negative | 41 | | 0.4 | 0.0206 | Negative |  |
| 92/17 (ET) | 0.067 | 0.002 | 1 | Negative | 42 | 0.458 | 1 | Negative | | 41.4 | | 0.360 | 0.0581 | Negative | 41.4 | | 0.173 | 0.0144 | Negative |  |
| 134/17 (K+T) | 1.174 | 0.02 | 0.0001 | **Positive** | 30.666 | 0.416 | 0.0008 | **Positive** | | 29.766 | | 0.251 | 0.0006 | **Positive** | 28.966 | | 0.378 | 0.0004 | **Positive** |  |
| 148/17 (L+T) | 1.178 | 0.004 | 0 | **Positive** | 26 | 0.264 | 0.0002 | **Positive** | | 25.166 | | 0.288 | 0 | **Positive** | 24.1 | | 0.173 | 0.0001 | **Positive** |  |
| 174/17(L+T) | 1.154 | 0.007 | 0 | **Positive** | 25 | 0.3 | 0.0002 | **Positive** | | 24.7 | | 0.2 | 0.0003 | **Positive** | 22.833 | | 0.493 | 0.0002 | **Positive** |  |
| 31/17 (L+T) | 0.091 | 0.009 | 0.0103 | Negative | 43.5 | 0.556 | 0.0173 | Negative | | 41.933 | | 0.208 | 0.0865 | Negative | 41.4 | | 0.36 | 0.0301 | Negative |  |
| 81/17 (L+T) | 0.094 | 0.014 | 0.03015 | Negative | 40.666 | 0.493 | 0.0539 | Negative | | 40.433 | | 0.416 | 0.0267 | Negative | 41.416 | | 0.381 | 0.0321 | Negative |  |
| 83/17 (L) | 0.090 | 0.014 | 0.0459 | Negative | 41.666 | 0.305 | 0.2889 | Negative | | 41.366 | | 0.568 | 0.0854 | Negative | 42.833 | | 0.208 | 0.5 | Negative |  |
| 147/17 (L+T) | 0.086 | 0.009 | 0.02515 | Negative | 42.333 | 0.416 | 0.3536 | Negative | | 42.733 | | 0.251 | 0.4471 | Negative | 42.833 | | 0.288 | 0.5 | Negative |  |
| 29/18 (ET) | 1.735 | 0.059 | 0.0002 | **Positive** | 16.166 | 0.288 | 0 | **Positive** | | 15.566 | | 0.513 | 0.0003 | **Positive** | 15.133 | | 0.152 | 0 | **Positive** |  |
| 106/18 (EC) | 1.952 | 0.042 | 0.0001 | **Positive** | 15.183 | 0.160 | 0 | **Positive** | | 15.166 | | 0.057 | 0 | **Positive** | 15.066 | | 0.115 | 0 | **Positive** |  |
| 108/18 (ET) | 1.491 | 0.049 | 0.0002 | **Positive** | 20.85 | 0.132 | 0.0001 | **Positive** | | 20.333 | | 0.288 | 0.0001 | **Positive** | 19.666 | | 0.288 | 0.0001 | **Positive** |  |
| 109/18 (ET) | 1.813 | 0.012 | 0 | **Positive** | 15.266 | 0.251 | 0.0001 | **Positive** | | 15.266 | | 0.251 | 0.0001 | **Positive** | 15.3 | | 0.1 | 0 | **Positive** |  |
| 116/18 (ET) | 1.407 | 0.0125 | 0 | **Positive** | 22.333 | 0.288 | 0.0002 | **Positive** | | 21.933 | | 0.152 | 0.0001 | **Positive** | 21.833 | | 0.208 | 0.0001 | **Positive** |  |
| 134/18 (ET) | 1.782 | 0.022 | 0 | **Positive** | 15.733 | 0.251 | 0.0001 | **Positive** | | 15.666 | | 0.321 | 0.0001 | **Positive** | 15.333 | | 0.288 | 0.0001 | **Positive** |  |
| 162/18 (EC) | 1.204 | 0.007 | 0 | **Positive** | 23.6 | 0.360 | 0.0003 | **Positive** | | 22.666 | | 0.288 | 0 | **Positive** | 22.266 | | 0.461 | 0.0002 | **Positive** |  |
| 163/18 (EC) | 1.486 | 0.028 | 0.0001 | **Positive** | 20.666 | 0.577 | 0.0003 | **Positive** | | 19.666 | | 0.305 | 0 | **Positive** | 19.2 | | 0.2 | 0.0001 | **Positive** |  |
| 215/18(ET) | 1.564 | 0.014 | 0 | **Positive** | 19.3 | 0.264 | 0.0001 | **Positive** | | 18.433 | | 0.416 | 0.0001 | **Positive** | 18 | | 0.1 | 0 | **Positive** |  |
| 269/18 (EC) | 1.200 | 0.003 | 0 | **Positive** | 23.6 | 0.36 | 0.0003 | **Positive** | | 23.366 | | 0.23 | 0.0001 | **Positive** | 22.433 | | 0.115 | 0.0001 | **Positive** |  |
| 311/18 (ET) | 1.234 | 0.006 | 0 | **Positive** | 25.033 | 0.55 | 0.0004 | **Positive** | | 25.1 | | 0.264 | 0.0001 | **Positive** | 24.666 | | 0.61 | 0 | **Positive** |  |
| 349/18 (ET) | 0.094 | 0.006 | 0.0157 | Negative | 41.166 | 0.152 | 0.0433 | Negative | | 42.333 | | 0.288 | 0.1403 | Negative | 42.9 | | 0.173 | 0.4069 | Negative |  |
| 33/19 (EC) | 0.094 | 0.006 | 0.023 | Negative | 41.8 | 0.2 | 0.2353 | Negative | | 41.633 | | 0.230 | 0.0273 | Negative | 41.233 | | 0.461 | 0.0296 | Negative |  |
| 34/19 (EC) | 1.235 | 0.016 | 0 | **Positive** | 21 | 0.3 | 0.0002 | **Positive** | | 21 | | 0.2 | 0.0001 | **Positive** | 19.733 | | 0.251 | 0.0001 | **Positive** |  |
| NC | 0.066 | 0.003 | - | Negative | 42 | 0.36 | - | Negative | | 42.8 | | 0.264 | - | Negative | 42.833 | | 0.288 | - | Negative |  |
